# Using Digital Mental Health and Measurement-Based Care With Youth Experiencing Suicidal Thoughts and Behaviors: Qualitative Study of Clinician Barriers and Facilitators

**DOI:** 10.2196/81505

**Published:** 2026-05-18

**Authors:** Lia Norman, Katherine Bright, Karina Pintson, Julia Hews-Girard, Adrijana D'Silva, Courtney Habina, Diana F Pricop, Geneca Henry, Sarah Daniel, Lauren Volcko, Katelyn Greer, Jason Gondziola, Leanne Stamp, Melanie Fersovitch, Karen Moskovic, Jessica Bradley, Haley M LaMonica, Ian Hickie, Frank Iorfino, David Johnson, Paul Arnold, Gina Dimitropoulos

**Affiliations:** 1 Department of Psychiatry, Cumming School of Medicine, University of Calgary, Calgary, AB, Canada; 2 Mathison Centre for Mental Health Research and Education, University of Calgary, Calgary, AB, Canada; 3 Faculty of Social Work, University of Calgary, MacKimmie Tower 413, 420 Campus Lane NW, Calgary, AB, T2N 1N4, Canada, 1 403 220 7332; 4 School of Nursing and Midwifery, Faculty of Health, Community, and Education, Mount Royal University, Calgary, Canada; 5 Faculty of Nursing, University of Calgary, Calgary, Canada; 6 Hotchkiss Brain Institute, University of Calgary, Calgary, AB, Canada; 7 Department of Pediatrics, Cumming School of Medicine, University of Calgary, Calgary, AB, Canada; 8 Brain and Mind Centre, The University of Sydney, Camperdown, Australia; 9 Maternal Newborn Child and Youth Strategic Clinical Network, Alberta Health Services, Calgary, Canada; 10 Alberta Children’s Hospital Research Institute, University of Calgary, Calgary, AB, Canada

**Keywords:** digital mental health, digital health, measurement-based care, mental health care, early intervention, mental health, suicide, suicidal thoughts and behaviors, youth, young adults, qualitative research, implementation science

## Abstract

**Background:**

Youth suicide is a public health crisis. In addition to suicide mortality, many youth experience and live with suicidal thoughts and behaviors (STBs). STBs have serious consequences for youth mental health and are associated with suicide. Despite recognition of the incidence and severity of STBs, barriers to accessing support are prevalent. Digital mental health (DMH) and digitally delivered measurement-based care (MBC) may improve access to treatment among youth and enhance clinical response to suicide risk and crises.

**Objective:**

This study aimed to explore clinician perceptions of the barriers and facilitators to using DMH and MBC with youth experiencing STBs.

**Methods:**

As part of a larger implementation science project, a DMH and MBC platform was implemented in youth-serving mental health service settings in communities across Alberta, Canada. The platform included a multidimensional assessment package and embedded an automated suicide escalation protocol that notified clinicians when youth reported STBs. In participating service settings, 32 interviews were conducted with clinicians using DMH and MBC. We used the Consolidated Framework for Implementation Research and theoretical thematic analysis to identify barriers, facilitators, and relevant themes.

**Results:**

Four overarching themes were identified that described barriers and facilitators to using DMH and MBC with youth experiencing STBs: (1) service setting and professional practice barriers, (2) DMH platform barriers, (3) service setting and professional practice facilitators, and (4) DMH platform facilitators. Clinicians’ recommendations were presented in alignment with the identified barrier themes.

**Conclusions:**

Given the association between STBs and suicide, attending to STBs is a critical component of suicide prevention. Our findings suggest that the assessment of STBs using DMH may address the youth suicide crisis by facilitating disclosure and rapid clinical action. Moreover, multidimensional assessment may reveal important information about STBs and risk factors, with advantages for personalized care and system-level improvement. Clinicians delivering DMH and MBC must be supported by applying their recommendations and continuously strategizing to mitigate barriers and leverage facilitators for sustained implementation.

## Introduction

### Background

Youth suicide is a public health crisis [1-4]. In Canada, suicide is the second leading cause of death among youth and young adults aged 15-34 years [5] and accounts for approximately 25% of deaths among youth aged 15-24 years [6]. A study of the epidemiological impact of youth suicide in countries with the highest human development index found that Canada has the third highest crude suicide mortality rate among youth in the 15-24 age range [7]. Each suicide signifies a young person who matters, years of potential life lost prematurely, and exerts a heartrending impact on families, friends, and communities [8]. Beyond suicide mortality, many youth experience and live with suicidal thoughts and behaviors (STBs) [5,9]. STBs comprise all facets of suicidality: active suicidal ideation (with intent to act), passive suicidal ideation (without intent to act), deliberate self-harm, preparatory acts (eg, planning suicide), and suicide attempts [10]. Youth aged 15-19 years exhibit the highest rates of urgent care visits and hospitalization related to self-harm in Canada [5]. Notably, STBs have serious and possibly debilitating consequences for youth’s physical and mental health and well-being [8].

Risk factors for STBs and suicide are heterogeneous, fluctuating, and interactive [4,5,11]. At the systemic and community level, residing in a remote or rural area [5,12], social and economic deprivation [3,5], natural disasters, and war [8,13,14] may elevate youth’s vulnerability. Other powerful systemic and community influences include experiences of marginalization [4], minoritization, discrimination, prejudice, loss of cultural identity [3], and racism [5]. For youth, interpersonal factors such as negative family and peer relationships, the death of a family member or friend [3], and peer victimization [3,15] are instrumental. Experiences of child maltreatment [16-18], witnessing intimate partner violence [3], gender-based violence [5,18], and other trauma also contribute to psychosocial stress and potential self-harm. Finally, individual factors interact with and are affected by broader systemic, community, and interpersonal dynamics [4,8]. Psychological distress [19] and feelings of worthlessness, self-loathing, isolation, and other emotions [3] are associated with youth STBs. Although not all youth experiencing STBs have a psychiatric disorder (eg, posttraumatic stress disorder), mental ill-health and comorbidities increase youth’s risk [10]. A personal history of STBs is a salient contributing factor to youth suicide [2,20]. Biologically, adolescence represents a period of vulnerability to developing STBs due to rapid neurodevelopmental and physical changes [4], limited cognitive control, and lower inhibition [1]. Isolation and disrupted routines during the COVID-19 pandemic interrupted typical adolescent development [4] and increased youth’s risk of suicidality [1,21,22] and suicide [1,4].

Due to pervasive social inequities, risk factors and related prevalence of STBs and suicide are not equally distributed across population groups [4,5,8]. Indigenous peoples (First Nations, Métis, and Inuit) in Canada experience risk factors including intergenerational trauma, marginalization, discrimination, racism, loss of land and culture, and differential access to resources [5,23-25] (eg, mental health services, housing, and clean water) as a consequence of historical and present colonialism and oppression [23,24]. Social and health disparities resulting from ongoing injustices contribute to elevated rates of suicide among Indigenous youth [24,26]; among Indigenous youth aged 15 to 24, the suicide rate is 5 to 6 times higher than that of non-Indigenous populations [23,27]. The suicide rate among Inuit youth in this age range is 11 times higher [23,27] and may be increasing among Inuit children aged 10-14 years [24]. However, Indigenous populations are diverse [25], and suicide is not a universal phenomenon in all Indigenous communities [23,25,28]. Community contextual factors (eg, environmental location and Indigenous governance in the community) and aspects of intersectional identity (eg, gender, sexual orientation, and social location) greatly affect risk factors for suicide [28].

Among other racialized populations, collective and individual experiences of marginalization, discrimination, racism [29,30], racial trauma [31], barriers to health resources [32], and other factors also contribute to suicide risk. There is a notable lack of race-based and ethnicity-based data [29,32] documenting suicide rates, and a lack of research to better understand unique pathways to suicidality among heterogeneous racialized communities and individuals in Canada [29]. Similarly, as gender identity and sexual orientation are not typically collected in death records, suicide rates among Canadian Two-Spirit, lesbian, gay, bisexual, transgender, queer, questioning, intersex, and asexual (2SLGBTQIA+) youth are unknown [5] or underreported. Existing data demonstrate that transgender and sexual minority youth are at higher risk of suicidal ideation and attempted suicide than their cisgender, heterosexual peers [33]. As with other historically and presently marginalized populations, heightened risk of STBs among 2SLGBTQIA+ youth occurs due to external inequities and influences, such as minority stress in various forms across societal and interpersonal levels [34-36] and potential victimization [37]. Experiences of STBs are again impacted by intersectionality and within- and between-group differences [33,35,37].

Despite recognition of the prevalence of STBs and suicide in youth, and the disproportionate burden in some communities, many youth struggling with STBs are unable to access appropriate support and treatment [10,38]. Barriers to accessing youth mental health care in Canada include limited resources in remote and rural areas, long waitlists, the cost of specialized services, a lack of support for transition-aged youth, stigma toward mental health concerns, and minimal or absent culturally competent care [39]. Among youth with access to health care systems, barriers to disclosing STBs are well documented. Many youth experience concerns about the confidentiality of their disclosure [40-42], fears of reexperiencing past harmful reactions to disclosure [42], and anxiety about hospitalization [41]. Feelings of hopelessness [40], isolation [3], and shame [41], along with fears of stigma [3], may also prevent youth from talking about STBs. Notably, 2 studies highlight that youth may not disclose STBs to clinicians if clinicians do not directly ask questions about suicidality [40,42]. A lack of clarity around roles and responsibilities for responding to suicide risk, worries about community follow-up (eg, when youth are sent to hospital but not admitted), limited time, competing priorities, and the need for training [43] may deter clinicians from initiating conversations about STBs with youth.

### Digital Mental Health and Measurement-Based Care for Youth Experiencing STBs: Overview of Existing Evidence

Considering the barriers to youth mental health care and the sensitivity around discussing STBs, tools that improve youth’s access to treatment and address STBs are urgently needed. Digital mental health (DMH) includes various web- and mobile-based apps that may be used to self-manage mental health concerns [38] or used in conjunction with clinician care [19,38,44,45]. Specific to STBs, DMH modalities such as telemedicine [44], videos, games, apps, supportive text messaging [38], and wearable devices [20] leverage technology to support youth experiencing ongoing suicidality and youth in acute crises. DMH may reduce barriers to treatment access by improving accessibility irrespective of youth’s geographic location [19,46], eliminating waitlists, providing low- or no-cost care options, expediting access to information [45], and increasing anonymity [46]. Some research indicates that DMH may be accessible for communities experiencing systemic marginalization, provided that barriers to access are addressed, and DMH is co-designed with the community it intends to support [47-49]. Co-design enables DMH to recognize the unique experiences of diverse groups of youth, meet their self-identified needs [48,49], and improve cultural relevance and relatability [47]. Importantly, youth perceive technologies as acceptable [44,50] and even preferable [51,52] means of crisis management and intervention. Youth who have not previously shared their STBs [53] and youth with difficulties communicating verbally [52,54] may find that DMH facilitates communication and disclosure about STBs with clinicians. Furthermore, DMH can play a role in safety planning by providing psychoeducation, teaching coping strategies [38], connecting youth to crisis resources and emergency contacts (eg, clinicians and caregivers) [38,55], and enabling rapid clinical response [53].

A significant advantage of DMH is its ability to deliver assessments of STBs and subsequently facilitate measurement-based care (MBC) [56]. In mental health care, MBC involves integrating continuous measurement of a person’s outcomes across one or multiple health domains into their treatment. The digital delivery of MBC through DMH apps and websites, alongside traditional services, offers a promising way to identify youth experiencing STBs and detect changes in their suicidality over time [19,53]. Clinicians monitoring youth’s MBC assessments in DMH who are alerted of suicide risk can respond by initiating contact with youth, developing a safety plan, and coordinating and escalating youth’s level of care. A recent mixed methods study reported that DMH and MBC improved clinicians’ response to suicide risk [57]. Given that the direct assessment of STBs is associated with a higher likelihood of disclosure [41], and that youth express wanting clinicians to initiate difficult conversations about STBs [40], DMH and MBC hold potential to improve awareness of and communication about suicidality for both youth and clinicians.

A small number of studies have documented clinician concerns about using DMH and MBC with youth. For instance, responding to crises may be outside clinicians’ scope of practice [58]—particularly if crisis alerts are received outside standard working hours [58-61]. This contributes to apprehension about liability due to potential delays in responding to youth at risk of suicide [58,59]. Additionally, clinicians report having limited time to implement digital tools due to low staffing availability [45], heavy workloads [58,60], and a lack of dedicated time in their schedules to review online assessments [58]. The remote nature of DMH raises concerns about clinicians’ lack of control to manage crises in person (eg, being unable to direct the patient to hospital) [46]. For these reasons, implementing DMH and MBC to support youth may be challenging without changes to staffing and roles [60].

Literature exploring the perspectives of clinicians responsible for using DMH and MBC with youth experiencing STBs appears limited. While review papers summarize various types of DMH for use with this population [20,38,44,51,52], and studies with youth [19,50,53,55] and caregiver participants [55] provide valuable insights, there is a lack of implementation research specific to clinicians and digitally delivered MBC. We identified 9 relevant studies that included clinician perceptions: 2 investigated other types of DMH [45,46], 3 examined monitoring tools similar to MBC [54,61,62], and 2 considered DMH and/or MBC more broadly [57,60]. Two studies were previously conducted by our research and implementation team in preimplementation project phases [58,59].

### Study Purpose

Suicide is preventable [8], and suicide prevention is a global imperative recognized by the World Health Organization (WHO) [8,63], the United Nations Sustainable Development Goals [63], and the Government of Canada [5]. Suicide prevention involves multisectoral collaboration [8], empowering communities, youth, clinicians [5], and families with mental health resources, as well as early identification and intervention [8]. Implementing DMH and MBC in youth-serving settings may support suicide prevention efforts; however, the inclusion of clinician perspectives remains a gap in the literature. In-depth qualitative research exploring postimplementation clinician perspectives is necessary to determine what clinicians need to use DMH and MBC effectively and relationally with youth. This study therefore aimed to explore clinicians’ perceptions and experiences through the following research question: “What are the barriers and facilitators to using DMH and MBC with youth experiencing STBs?”

This study is part of a larger mixed methods implementation science project evaluating the implementation of a DMH and MBC platform in youth mental health service settings in rural and urban communities across Alberta. The larger project, implementation process, and platform are described in previous publications [58,59,64-66]. Among several outcome objectives, the project is assessing the impact of implementation on emergency department use, youth mental health, and suicide attempts. This study extends our prior work by presenting data collected postimplementation, focusing explicitly on youth experiencing STBs, and using a well-established implementation science framework to identify barriers, facilitators, and clinician recommendations specific to this topic.

In this paper, unless specifically referring to MBC, we hereinafter refer to DMH and MBC collectively as “DMH.”

## Methods

### The Innowell DMH Platform: Description and Functions for Youth Experiencing STBs

The Innowell platform (Innowell PTY Ltd) was developed in Australia through co-design with individuals with lived experience of mental illness and health care professionals [56,57,67,68]. It is web-based and contains a multidimensional assessment package of clinical questionnaires for 20 biopsychosocial domains. Youth complete the initial baseline package and can repeat all or selected questionnaires as needed. Once complete, results are presented in a dashboard with color-coded “health cards” that indicate the severity (low-high) of youth’s needs. Visual graphs that reflect improvement or decline over time are generated as questionnaires are retaken. In addition, relevant apps, e-tools, and community-specific resources are provided. The platform enables MBC and encourages collaboration by allowing shared access to youth data for youth and their clinicians. Clinicians can personalize care by monitoring results according to service setting procedures, exploring questionnaire responses with youth, and engaging youth in shared decision-making about their treatment.

Innowell includes a Suicidal Thoughts and Behaviors domain and embeds an automated suicide escalation protocol [56,67,68]. In the STBs domain, questions come from 2 evidence-based measures covering current and historical STBs and their intent (eg, suicidal ideation with or without a plan or attempt): the Suicidal Ideation Attributes Scale [69] and the Colombia-Suicide Severity Rating Scale [70]. Any expression of STBs in the questionnaire immediately triggers a pop-up notification with crisis helplines and community-specific crisis resources. If the severity of risk meets a threshold determined by the service setting, a notification is also sent to the youth’s clinician and any other support person monitoring their DMH profile. The notification remains “flagged” for clinicians until they have contacted the client to confirm their safety, develop a safety plan, book an appointment, or any other action deemed appropriate by the youth and clinicians. In this way, the protocol enables identification, monitoring, and rapid clinical response to suicide risk.

Service settings implementing Innowell developed procedures for responding to crises and advised clinicians not to check the platform or respond to notifications outside working hours. However, many clinicians continued to monitor the platform at other times.

### Service Settings and Participant Recruitment

Participating primary care networks (PCNs), specialized mental health services, schools, and community-based organizations in Alberta implemented the platform between 2021 and 2024. Implementation was facilitated by implementation leads located in participating communities who provided DMH education, training, and support to clinicians. Clinicians invited youth aged 15-24 years to use the platform as part of their care. To recruit clinicians with differing experiences with DMH, the research and implementation team invited clinicians who used the platform extensively, moderately, and minimally to participate.

### Data Collection

A semistructured interview guide was developed based on the research and implementation team’s expertise in implementation science and integrating DMH in clinical practice. Three topic areas were included in the guide: (1) implementation experience, which explored participants’ roles, their use of DMH, and barriers and facilitators to implementation; (2) impact, which considered the impact of DMH on workflows, supporting youth with acute care needs (including youth experiencing STBs), and youth engagement in treatment; and (3) sustainability, which addressed participants’ willingness to continue using DMH and recommended changes to facilitate sustained implementation. The semistructured approach allowed participants to respond to planned topics and questions, while offering opportunities for reflections related to STBs and other pertinent issues to occur at different times. This study’s topic was inductively derived from the content of the interviews.

Individual interviews were conducted online via Zoom (Zoom Communications, Inc) to accommodate participants’ various locations in Alberta, and facilitated by a senior member of the research team and one research assistant (RA). Both facilitators had no prior relationship with participants. The duration of interviews varied from 60 to 90 minutes. All interviews were recorded, transcribed, anonymized, and reviewed by the research team before data analysis to ensure their anonymity and accuracy.

### Data Analysis

#### Qualitative Descriptive Approach

This study followed a qualitative descriptive approach [71]. In contrast to qualitative methods involving recontextualization of the data [72], qualitative description seeks to explore the data using low-inference description that closely reflects participants’ own words about their perceptions and experiences [71]. Descriptive methods are well-suited to health research and intervention studies, as their straightforward documentation of why the intervention did or did not work, and what could improve, can be directly applied to create clinically meaningful change [73]. With consideration of our study’s purpose, the qualitative descriptive approach was chosen to provide a rich description of participant-identified barriers and facilitators to using DMH with youth experiencing STBs.

Qualitative description is adaptable and allows for several possible means of analyzing the data [73]. We conducted our analysis in three phases: (1) deductive coding using a recognized implementation science framework [74] to systematically organize the entirety of the data; (2) rating constructs (codes) to identify what constructs served as barriers and facilitators; and (3) conducting a theoretical thematic analysis [75] to identify patterns within the constructs, group constructs into themes, and provide an in-depth description of themes in response to the research question.

#### Analysis Phase 1: Deductive Coding Using the Consolidated Framework for Implementation Research

The Consolidated Framework for Implementation Research (CFIR) is a highly cited implementation science resource designed to assess determinants of innovation implementation from the perspectives of involved individuals and teams [74]. Deductive coding using the CFIR can identify factors influencing implementation related to the innovation itself, larger systems, the organization, individuals delivering and receiving the innovation, and implementation processes, and inform recommendations for sustained integration. Using the CFIR enhanced our adherence to low-inference qualitative description across all phases of analysis by providing a framework in which to situate and understand our data, thereby reducing bias and overinterpretation and improving consistency between coders.

The CFIR includes 5 domains to structure research data [74] (refer to Table 1; definitions are paraphrased for brevity and adapted for relevance to this study). Each domain contains constructs and subconstructs (39 in total) with definitions for use in coding that may be understood within the implementation context and adapted as necessary.

**Table 1.** Consolidated Framework for Implementation Research (CFIR) overarching domains (adapted from Damschroder et al [74]).

CFIR^a^ domain	Paraphrased description
Innovation	This domain includes factors related to the innovation being implemented (DMH^b^). CFIR constructs consider the innovation’s evidence-base, design, adaptability, complexity, cost, and other innovation features that may influence implementation.
Outer Setting	This domain includes factors related to the larger setting and systems in which the inner setting is situated (eg, health care system and legal system). CFIR constructs consider local conditions, partnerships, laws, policies, financing, and other external conditions that may influence implementation.
Inner Setting	This domain includes factors related to the setting where the innovation is directly implemented (youth-serving mental health service settings). CFIR constructs consider the inner setting’s structure, staffing, culture, and other internal factors that may influence implementation. Constructs also consider whether the inner setting is accepting of change, if implementation is prioritized, and the compatibility of the innovation with inner setting processes.
Individuals	This domain includes factors related to individuals involved in implementation (clinicians and youth). CFIR constructs consider the capability, opportunity, and motivation of innovation deliverers and recipients to implement and receive the innovation, and whether the innovation meets deliverer and recipient needs.
Implementation Process	This domain includes factors related to the process of implementing the innovation (DMH). CFIR constructs consider initial assessments of deliverer and recipient needs, planning, strategizing, and collaborating during implementation, engaging recipients, and other processes required to implement the innovation.

a CFIR: Consolidated Framework for Implementation Research.

b DMH: digital mental health.

Before coding, reviewed transcripts were uploaded to a qualitative data analysis platform (Taguette [Rampin]) [76]. The research team familiarized themselves with the data by reading each transcript and memoing their observations for team discussion. Transcripts were then divided between 2 RAs and independently coded using the CFIR. RAs and the research team met weekly to share their progress, discuss questions, and resolve uncoded quotes that RAs sought feedback on. If disagreements about coding arose, the team referred to the CFIR construct definitions for clarification.

The final step involved creating a copy of the larger codebook and removing all constructs that did not contain quotes related to STBs. Within the remaining constructs, any quotes with unrelated content were removed to ensure that irrelevant topics were excluded from the second phase of analysis. Domains and constructs deemed applicable to this study are defined in the “Results” section.

#### Analysis Phase 2: Construct Rating—Assessment of Barriers and Facilitators

In the second phase, constructs that contained quotes related to STBs were rated using the CFIR Index Manual for Administration and Scoring [77]. The CFIR Index is a practical tool for research and quality improvement designed to classify CFIR constructs as barriers and facilitators to implementation and to assess the influence, valence, and strength of the constructs. In our study, we followed the step-by-step process outlined in the manual to identify constructs as barriers or facilitators to using DMH with youth experiencing STBs.

RAs first rated individual quotes as a negative (-), positive (+), or mixed (X) factor (containing both negative and positive sentiments). Using a 5-point Likert scale, RAs then applied a number between −2 and +2 to determine the strength of the factor’s influence on implementation. Most quotes related to STBs were clearly strong barriers or facilitators and rated accordingly as −2 or +2. To establish the construct’s overall rating as a barrier or facilitator, RAs observed the distribution of rated quotes and rated each construct according to the majority.

#### Analysis Phase 3: Theoretical Thematic Analysis

After rating relevant constructs, we conducted a theoretical thematic analysis as described by Braun and Clarke [75]. Theoretical thematic analysis is a deductive method of identifying and describing patterns in the data by applying preexisting theories or frameworks to answer a research question in detail. Guided by the CFIR, completed phases of deductive coding and construct rating produced barrier and facilitator constructs that acted as the framework for interpreting the data and clustering constructs into themes [78]. We followed Braun and Clarke’s [75] six-stage iterative analytic process [78] to explore data related to clinicians’ perceptions and experiences of using DMH with youth experiencing STBs, contained within relevant constructs. As with previous phases, the theoretical thematic analysis remained focused on observable details in the data in alignment with the qualitative descriptive approach [71].

In the first stage, RAs refamiliarized themselves with the data by rereading coded transcripts with the research question in mind and writing memos about relevant patterns. The second stage, coding, was previously completed. In the third stage, RAs examined the constructs and their associated quotes and began to combine them into larger patterns, themes, and subthemes. This involved considering what “central organizing concept” [78] was shared by each cluster of constructs. Developing a clear understanding of what constructs and ideas fit within a particular theme led to identifying more distinct groupings. Due to the CFIR’s broad definitions of constructs, some constructs contained multiple ideas and were therefore included in more than one theme. RAs ensured that ideas did not overlap between themes—despite the interconnectedness of the data overall. Subthemes captured distinct concepts but shared the same underlying concept as the theme.

In the fourth stage, themes were reviewed to confirm their central organizing concepts, ensure their relevance to the research question, and check that no key ideas in the dataset were missed. This step involved examining whether the themes told an accurate and meaningful story about the data. Finally, the fifth and sixth stages entailed defining and naming the themes and producing the paper. Exemplar participant quotes were thoughtfully selected to illustrate specific impressions in theme titles and descriptions.

##### Positionality

The research and implementation team includes researchers, clinicians, and clinician-researchers at various stages in their careers and with expertise in diverse disciplines (social work, psychology, psychiatry, nursing, medicine, education, and software design and development). It is vital to acknowledge that the topic of STBs and suicide is deeply significant to the authors. Some authors carry personal lived experience with STBs, have lost friends to suicide, have worked as clinicians with youth who completed suicide, and have worked in mental health advocacy alongside parents and caregivers who lost their children to suicide. Others have worked as researchers and facilitators with youth experiencing suicidality. These experiences have shaped our perspectives on life—and inevitably impacted the way we perceived and interacted with the data. We engaged in reflexivity by recognizing our unique perspectives, partaking in group discussions so that multiple perspectives were reflected in the research, and continuously referring to the data to ensure that participants’ voices remained central to the analysis.

##### Trustworthiness and Authenticity

This study adheres to the 5 standards used to assess trustworthiness and authenticity in qualitative research: objectivity, dependability, credibility, transferability, and application [71]. Objectivity was addressed by describing our methods in detail, recognizing potential biases, and reporting our positionality. To achieve dependability, we standardized data collection by using the same interviewer and interview guide across interviews, determining data analysis procedures based on the research question, and using a credible theoretical framework (the CFIR) to guide the analysis and remain close to the data. Researcher triangulation was achieved by 2 RAs independently coding the transcripts and subsequently discussing their findings with the larger team. To assess credibility, findings were shared with implementation leads to confirm that the analysis reflected their experiences with implementing DMH and engaging with clinicians using the platform. A discussion of transferability and generalizability of the findings is included in the “Strengths and Limitations” subheading. Finally, we considered the application of the study by presenting clinician and researcher recommendations for addressing the barriers to using DMH with youth experiencing STBs (refer to “Results” section and “Practical Implications and Researcher Recommendations” subheading).

### Ethical Considerations

This study was approved by the University of Calgary Research Ethics Board (REB20-1137). Potential participants were informed about the study purpose and procedures, that participation was voluntary, and that they could withdraw at any time. Before participation, all participants provided written informed consent and completed a demographic survey via REDCap (Research Electronic Data Capture; Vanderbilt University) secure software hosted at the University of Calgary. All identifying information was omitted from the paper to protect participants’ privacy and confidentiality. Monetary compensation was not provided because participation in the project entailed implementing DMH within the scope of participants’ regular duties.

## Results

### Participant Characteristics

Clinicians (N=32) from youth-serving mental health service settings participated in interviews: 17 from specialized mental health services, 9 from schools and community-based organizations, and 6 from PCNs. A majority were from rural communities (n=21, 66%). Participants occupied varying professions, including psychology and counseling (n=14, 44%), social work (n=11, 34%), and other roles (n=7, 22%). Most participants had worked for their service setting for ≤5 years (n=19, 59%). A majority worked full-time (n=25, 78%) and had graduate-level education (n=20, 63%). Participants’ ages ranged from 20‐29 to 50‐69 years. Most participants reported their assigned sex as female (n=25, 78%) and most identified as women (n=24, 75%). A majority were heterosexual (n=23, 72%). Just over half of participants had European ethnic origins (n=18, 56%), and others reported various ethnic origins (n=13, 41%). Several variables were collapsed into “Other” categories due to low numbers to protect participants’ identities. Full participant demographics are provided in Table 2.

**Table 2.** Participant demographics (N=32).

Variable	Value, n (%)
Community setting	
Rural community	21 (66)
Urban community	11 (34)
Service setting	
Specialized mental health services	17 (53)
Schools and community-based organizations	9 (28)
Primary care networks	6 (19)
Profession	
Psychology or counseling	14 (44)
Social work	11 (34)
Other	6 (19)
Missing	1 (3)
Length of time with organization	
<1-5 years	19 (59)
≥6 years	12 (38)
Missing	1 (3)
Full- or part-time work	
Full time	25 (78)
Part time	6 (19)
Missing	1 (3)
Education	
Graduate school	20 (63)
Bachelor’s degree	8 (25)
Other	3 (9)
Missing	1 (3)
Age (years)^a^	
20‐29	8 (25)
40‐49	8 (25)
30‐39	7 (22)
50‐69	6 (19)
Not reported	2 (6)
Missing	1 (3)
Assigned sex	
Female	25 (78)
Male	5 (16)
Missing	2 (6)
Gender identity	
Woman	24 (75)
Man	5 (16)
Missing	3 (9)
Sexual orientation	
Heterosexual	23 (72)
Other	8 (25)
Missing	1 (3)
Ethnicity	
European origins	18 (56)
Other	13 (41)
Missing	1 (3)

a Some variables were collapsed into an “Other” category due to low numbers. Contents of the “Other” categories are not reported to protect participants’ identities. Not reported age range: during data cleaning, we identified inconsistencies between some participants’ reported age (eg, ≤19 years) and education level (eg, graduate school). As such combinations were not feasible, these cases (n=2) were not reported to maintain data integrity. The extent of clinicians’ platform use is not reported to protect participants’ identities.

### Theoretical Thematic Analysis

#### Overview

The CFIR delineated barriers and facilitators to using DMH with youth experiencing STBs across all 5 CFIR domains: Innovation, Outer Setting, Inner Setting, Individuals, and Implementation Process. This reflects the salience of the matter in connection with participants’ service settings, professional practice, and DMH. Rated constructs that informed the theoretical thematic analysis are defined in Table 3.

**Table 3.** Synthesis of results.

Theme and subtheme^a^	CFIR^b^ domain and construct included in theme, with CFIR definitions
1: Service Setting and Professional Practice Barriers	
1.1: Service setting structure does not support immediate crisis response	Inner Setting: Structural Characteristics—Work Infrastructure (includes factors related to staffing and staff roles) Inner Setting: Compatibility (includes factors related to the compatibility of the innovation with existing inner setting processes) Individuals: Innovation Deliverers—Opportunity (includes factors related to the capacity, availability, and power of deliverers to effectively implement the innovation) Individuals: Innovation Deliverers—Motivation (includes factors related to deliverers’ motivation to implement and sustain use of the innovation)
1.2: DMH^c^ incites tension between clinicians’ responsiveness and burden of responsibility	Outer Setting: Policies and Laws (includes factors related to laws, regulations, and professional guidelines that may affect implementation; eg, mandatory reporting laws and code of ethics) Inner Setting: Compatibility (includes factors related to the compatibility of the innovation with existing inner setting processes) Individuals: Innovation Deliverers—Opportunity (includes factors related to the capacity, availability, and power of deliverers to effectively implement the innovation) Individuals: Innovation Deliverers—Motivation (includes factors related to deliverers’ motivation to implement and sustain use of the innovation)
1.3: Clinicians express nuanced concerns about youth who report STBs^d^ in DMH	Implementation Process: Assessing Needs—Innovation Recipients (includes factors related to assessing the needs of recipients to inform implementation) Individuals: Innovation Deliverers—Motivation (includes factors related to deliverers’ motivation to implement and sustain use of the innovation) Individuals: Innovation Deliverers—Opportunity (includes factors related to the capacity, availability, and power of deliverers to effectively implement the innovation)
2: DMH Platform Barriers	
2.1: Youth struggle to interpret the DMH STBs assessment and cope with results	Innovation: Evidence-base (includes factors related to existing evidence to support use of the innovation) Innovation: Complexity (includes factors related to the complexity of the innovation) Innovation: Design (includes factors related to the design of the innovation) Individuals: Innovation Recipients—Capability (includes factors related to recipients’ ability and knowledge to effectively use the innovation)
2.2: Continuous DMH crisis notifications for youth with persistent or past STBs are unhelpful	Innovation: Design (includes factors related to the design of the innovation) Implementation Process: Assessing Needs—Innovation Recipients (includes factors related to assessing the needs of recipients to inform implementation)
3: Service Setting and Professional Practice Facilitators	
3.1: DMH supports the therapeutic relationship between clinicians and youth	Innovation: Relative Advantage (includes factors related to whether the innovation enhances existing practice and processes) Inner Setting: Culture—Recipient-centeredness (includes factors related to the inner setting’s commitment to supporting innovation recipients) Inner Setting: Mission Alignment (includes factors related to whether the innovation aligns with the inner setting’s mission and purpose) Implementation Process: Teaming (includes factors related to team collaboration and coordination during implementation)
4: DMH Platform Facilitators	
4.1: DMH enables disclosure, identification, and early intervention	Innovation: Relative Advantage (includes factors related to whether the innovation enhances existing practice and processes) Individuals: Innovation Recipients—Need (includes factors related to whether recipients have needs that the innovation could address)

a All domains, constructs, and subconstructs are from Damschroder et al [74]. Definitions are paraphrased for brevity and adapted for relevance to this study.

b CFIR: Consolidated Framework for Implementation Research.

c DMH: digital mental health.

d STB: suicidal thoughts and behaviors.

During theming, we noted that service setting and professional practice factors were greatly interconnected. Four overarching themes were therefore identified that describe (1) service setting and professional practice barriers, (2) DMH platform barriers, (3) service setting and professional practice facilitators, and (4) DMH platform facilitators (refer to Table 3). Relevant CFIR constructs are noted in *italics* in each theme description.

#### Theme 1: Service Setting and Professional Practice Barriers

##### Overview

Theme 1 explores barriers to using DMH with youth experiencing STBs that occur in the larger organization and within clinicians’ daily practice. This includes subtheme 1.1 (service setting structure does not support immediate crisis response), subtheme 1.2 (DMH incites tension between clinicians’ responsiveness and burden of responsibility), and subtheme 1.3 (clinicians express nuanced concerns about youth who report STBs in DMH).

##### Subtheme 1.1: “Kids Don’t Just Go Into Crisis Monday to Friday, 8:30 AM to 4:30 PM”: Service Setting Structure Does Not Support Immediate Crisis Response

In most interviews, the structure and purpose of service settings as “not a crisis clinic” (P29) was a principal factor hindering clinicians’ ability and *motivation* to use DMH with youth experiencing STBs. This service setting barrier affected participants’ overall experience with DMH implementation and contributed to individual-level hurdles to integrating MBC specifically. Although DMH is accessible 24/7 and youth can complete the STBs domain at any time, participants questioned the *compatibility* of DMH that incorporates crisis notifications with their service settings and practice due to restricted clinic hours. “Time limitations” (P12) led to delayed responses to youth who scored high for STBs after clinic hours, on weekends, and during participants’ holidays. One participant captured the stress shared by many clinicians: “It’s terrible when you come to work and you look [at DMH] and see that they’ve been suicidal since Friday night, and now it’s Monday or Tuesday…” (P09). Despite knowing that DMH provides immediate access to crisis resources, including crisis helplines and prompts to visit emergency services, participants felt uneasy with the situation being outside their control and being unable to guarantee that youth would receive timely and appropriate support.

Service setting structure and purpose also related to clinicians’ *opportunity* to integrate DMH and crisis response into their daily workflows and processes. For many participants, DMH was considered “an unfair ask of clinicians, who don’t have flexibility in their schedule” (P29) to check and respond to crisis notifications during working hours. Concerns about clinicians’ limited availability were frequently tied to concerns that they may be unable to fulfill their existing roles and responsibilities if using DMH. One participant stated:

*What happens when we get that suicidal flag? Then what? I have a full day booked with 20 patients and I don’t have time to cold call this person and make sure they’re doing well…*

[P22]

In the context of their *work infrastructure*, such as task distribution and staffing levels, several participants consequently struggled to see DMH as a viable means to support youth in crisis.

##### Subtheme 1.2: “You Should Be Able to Leave Work at Work”: DMH Incites Tension Between Clinicians’ Responsiveness and Burden of Responsibility

Clinicians’ internal conflict about receiving crisis notifications outside working hours was a significant consideration related to the *compatibility* of DMH with their practice. This involved a tension between their compassion and desire to meet youth’s urgent needs, and feeling emotionally burdened by crises that occurred in their personal time. One participant articulated consequences to clinicians’ mental health:

*I can’t ignore it. I don’t care if it’s a day off, I’m going to call that person. I don’t mind doing that, because I care about my people… But for my own mental health, there were some nights where you get an email […], and I’m sitting there stressing about this individual who has just flagged as high-risk for suicide*.

[P04]

Several clinicians also noted ongoing prospective anxiety about crises arising outside work, regardless of whether this transpired in reality. One participant wondered, “…in the middle of the night, are we supposed to respond? […] I would probably not sleep until I got to talk to them” (P14), and another expressed, “If something did happen where they completed [suicide], it would break your heart, that we weren’t there for them” (P09). In some cases, feelings of burden negatively impacted clinicians’ general perceptions of DMH and their *motivation* to continue using it. Some participants had removed or wanted to eliminate their work email from their phone to set explicit boundaries between their practice and private lives.

Concerning clinicians’ inner tension around DMH, participants shared additional worries about *policies and laws* dictating the need to attend to youth experiencing STBs within a set timeframe. Clinicians’ lack of *opportunity* for crisis response meant that some participants were wary to “take on liability that I know I can’t attend to” (P29). For one participant, the potential implications of not responding arose in practice and created great discomfort. They described a situation in which a client was assessed as stable during their appointment, but later scored high for STBs in DMH:

*…it was a question of liability. I believe it was a Friday, I had very little time to follow up, and the question was ‘how’ and ‘what if’ […]. So, it was a real moral dilemma for me of, how do I follow up now that I have this information, now that it’s in my hands? […] I didn’t appreciate that part of my role is somehow to follow up and always be monitoring if they’re scoring high on risk […]. Ultimately, we're still somewhat responsible if we do have information in between sessions if the risk changes, and I wasn't comfortable with that*.

[P20]

Due to their apprehension about liability, some participants desired “…some version of this program [DMH] where it feels less like my responsibility to check in on, to be a part of […] …something that alleviates my level, or flattens and equalizes my level of responsibility with the client” (P31).

##### Subtheme 1.3: “How to Respond in an Effective, Timely, Equitable Way”: Clinicians Express Nuanced Concerns About Youth Who Report STBs in DMH

As clinicians engaged in *assessing the needs* of youth to guide DMH implementation, they encountered unique concerns about youth who experience STBs self-reporting their symptoms. Some participants had worked with youth that they characterized as “underreporters” (P01) who revised their responses in the DMH STBs domain or stopped using DMH entirely after being flagged as high-risk. Despite prefacing that the purpose of crisis notifications is to confirm youth’s immediate safety and engage in safety planning, some youth were “not very keen” (P16) about the follow-up process and “hesitant to go back” (P19) to using DMH. Participants observed that youth may minimize the severity of their STBs due to discomfort with seeing their results or fears that their caregivers would be notified. This was understood as limiting the purpose and practicality of using DMH with certain youth.

Beyond underreporting, a more prevalent issue affecting clinicians’ *motivation* to continue offering DMH was their concern that some youth “overreported” their STBs to receive a quicker response from clinicians and a higher level of care to validate their experiences. Some participants felt that youth used DMH “like a bat signal” (P24) to communicate in-the-moment feelings of distress, which they did not perceive as genuine crises. One participant explained:

*If they plot it [STBs] in the moment in which they have acuity or crisis, then it becomes real. […] Something about the fact that it now exists on that platform […], it almost enhances or amplifies what it means. Because it’s available in that moment and there’s that protective element of anonymity, people just shoot for the stars, so to speak*.

[P31]

“Overreporting” was noted as common among youth with complex diagnoses and acute care needs, who experience “a lower level of tolerance for suicidality […] because of the structure of their thoughts” (P01). Importantly, one participant eloquently stated the “nuances” (P16) of why youth may recurrently score high for STBs in DMH:

*…if [DMH] is the only thing that they have, that might be the only place that they’re comfortable informing that ‘I am in crisis’. […] If it was just left up to the platform itself… It’s hard for youth to understand, and it’s hard for them to not show that they’re in crisis as well, if that makes sense*.

[P16]

Their sentiment illustrates that DMH may sincerely reflect the perspectives of some youth, and that youth may use DMH as a cry for help.

Considering their therapeutic practice and aims, participants saw that youth reporting STBs in DMH—and the follow-up process—may inadvertently oppose distress tolerance practices learned in treatment and reinforce ineffective coping. Most participants were unsure how to manage frequent crisis notifications that they perceived did not represent youth’s true level of distress, and had limited *opportunity* to do so. One participant voiced the uncertainty shared by other clinicians:

*I felt really torn… […]. I really struggled with how to respond in an effective, timely, equitable way, while also establishing and modelling healthy attachment and healthy boundaries. I still don’t know the answer. […] I don’t want to ignore the button, and then one day it is a crisis and they do need immediate support […]. But, I have a full caseload now and I don’t necessarily have time to take away from another client in order to respond to what I’m thinking is a crisis, but it isn’t a crisis*.

[P07]

Collectively, clinicians faced multilayered concerns when integrating DMH into their practice with youth with complex needs who have varying ways of communicating their suicidality and need for support.

### Theme 2: DMH Platform Barriers

#### Overview

Theme 2 explores barriers to using DMH with youth experiencing STBs that relate to qualities of DMH itself. This includes subtheme 2.1 (youth struggle to interpret the DMH STBs domain and cope with results) and subtheme 2.2 (continuous DMH crisis notifications for youth with persistent or past STBs are unhelpful).

#### Subtheme 2.1: “This Doesn’t Feel Safe”: Youth Struggle to Interpret the DMH STBs Domain and Cope With Results

A DMH barrier for clinicians was the *design* and *complexity* of the STBs domain and its associated assessment questions. Based on their experience and feedback from youth, participants remarked that questions about STBs were poorly worded and contained “gray areas” (P30) that could be interpreted in multiple ways. One participant shared,

*…a good example would be […], have you ever had a suicidal thought? ‘Yes’. And then they’re like, but it was 20 years ago, or 10 years […], it was a long time ago. When it’s not relevant anymore, it’s still relevant on that survey*.

[P30]

Several participants commented that youth’s *capability* to comprehend the questions and accurately reflect their current STBs in their responses was subsequently limited by the lack of context associated with some of the wording. Moreover, concerns emerged related to youth’s capacity to understand the overall purpose and context of the STBs domain—and the necessary follow-up that may occur as a result of their responses. One participant spoke to their experience working with young clients:

*…a lot of children […], they do make comments about wanting to end their life or feelings of wanting to die. That can be for a multitude of reasons. It’s not always suicidal ideation… It can come from wishing to be with a family member that’s in heaven. It could be a way of expressing, ‘I just don’t want to feel angry anymore’, or ‘I can’t deal with this really big feeling’ […]. So, I worry about children being on the platform and not understanding the context…*

[P21]

Given the lack of clarity, some participants questioned the STBs domain’s effectiveness, accuracy, accessibility, and other *evidence-base* factors. One participant expressed, “I’m going to be very blunt; I don’t trust the suicide scale on the platform” (P20), and another said, “I don’t think that the suicide screen available on the platform is efficient” (P21).

Another concern among clinicians was youth’s *capability* to cope with reading and seeing the visual of their STBs assessment results. If youth scored high for STBs, participants worried about how youth might “interpret or sit with this information, especially if the client is presenting with challenges to stress tolerance with new information” (P25). One participant captured clinicians’ fears about youth completing the STBs domain by themselves:

*…expecting them to do all of that on their own, without any co-regulation, without any coaching… […]. This doesn’t feel safe, especially if they are suicidal youth, that doesn’t cope well*.

[P17]

Accordingly, some participants needed to work through DMH with youth to ensure their safety during and after completing the questionnaire. This was perceived negatively due to taking additional time from youth’s sessions and limiting clients’ self-determination to discuss issues that matter to them.

#### Subtheme 2.2: “Pigeonholed and Stuck There”: Continuous DMH Crisis Notifications for Youth With Persistent or Past STBs Are Unhelpful

In discussing the DMH automated suicide escalation protocol, many clinicians mentioned that the crisis notifications were “very sensitive for our population” (P24). Participants referred to the prevalence of persistent suicidality among their clients to contextualize their concerns with the *design* of the system. If youth experiencing ongoing STBs completed the STBs domain on a regular basis, clinicians were frequently notified about youth whom they were already aware of and closely monitoring. Some participants found this unnecessary and felt it did not add value to their practice. With regard to *assessing the needs* of youth with persistent STBs, participants observed that youth themselves were discouraged by being consistently flagged as “permanent high-risk” (P06). One participant explained:

*I had some youth who got very frustrated that it always flagged, even if they weren’t actively suicidal […]. The patients who have chronic suicidal ideation, I feel like they were pigeonholed and stuck there, whereas they were making incredible gains in a lot of ways, but that portion of the platform didn’t depict that*.

[P04]

Another participant shared their understanding of youth’s thought process: “It was like, ‘this is something I go through. I’m not actually gonna do it. I actually don’t want to die, right now’” (P24). Regardless of how much time had passed, youth with personal histories of STBs (eg, a past suicidal episode) who disclosed this in DMH also triggered repeated crisis notifications for clinicians.

A related barrier concerning the *design* of the protocol was clinicians’ perception that continuous crisis notifications impeded their delivery of strengths-based therapy. Some participants found the automated protocol to be stigmatizing, and considered this “…a barrier for the client, but as well as for me as a practitioner—I don’t necessarily want to put my client in that position” (P06). Crisis notifications further distracted from other therapeutic goals that were part of youth’s treatment plan. One participant described their position:

*…when we’re flagging, then all of a sudden we’re re-diverting to that […]. That becomes the focus, rather than, how do we actually go backwards and look at resiliency, and how to help people grow and develop and work through things. We’re always talking about the end, fearful thing…*

[P17]

Although participants recognized the necessity of addressing suicidality, they highlighted that working from a strengths-based approach can underlie and support this broader intent.

### Theme 3: Service Setting and Professional Practice Facilitators

#### Overview

Theme 3 explores facilitators to using DMH with youth experiencing STBs that occur in the larger organization and within clinicians’ daily practice. This includes subtheme 3.1 (DMH supports the therapeutic relationship between clinicians and youth).

#### Subtheme 3.1: “You’re Not Alone”: DMH Supports the Therapeutic Relationship Between Clinicians and Youth

The ability for DMH to facilitate the therapeutic alliance was seen as a significant *relative advantage* of DMH for clinicians’ practice, compared with traditional services alone. Across the interviews, participants consistently said that youth using DMH and clinicians’ access to their results opened the door to important conversations about suicidality that may not have occurred otherwise. Participants felt that their reaching out to discuss youth’s STBs was “validating for the clients” (P11) and enabled youth to “feel like they get to tell their own story—I think it made them feel like somebody gave a darn about them” (P04). When clinicians received a crisis notification, most participants found that the follow-up process also had a profoundly positive effect on their connection with youth. One participant shared their impactful experience responding to youth in crisis:

*As a practitioner who is very relationship-based, to be able to reach out to somebody when they have identified they’re in need, and to do it quickly, I just think it’s such a gift […]. To be able to reach out to that person and say, ‘you’re not alone, these are the resources we have, we’re here for you’… I can only imagine being on the receiving end […]. When I think of a youth cohort, you’re often feeling unseen and unheard, and not valued or validated. For someone to be able to reach out in a time like that, I think is a huge benefit of the application. […] I feel like this tool, for me… It’s been like the magic wand that has allowed me to have a different kind of relationship*.

[P18]

Notably, some participants noticed that following up about youth’s STBs was particularly powerful for youth who struggle to trust adults and clinicians. Responding to crisis notifications demonstrated clinicians’ reliability and responsiveness to youth. One participant stated:

*I think there was something pretty powerful of, I tell you that I’m going to get this email, and I do actually follow through with following up with you. I think that did a lot for the relationship… […] They appreciated that an adult actually did do what they say they’re going to do*.

[P04]

Together with one-to-one relationships, DMH aided in cultivating a positive relationship among youth and the service setting at large. Participants provided numerous examples of *teaming* in which their team was able to “reach and wrap services around” (P18) youth experiencing STBs. For instance, one participant’s colleagues rapidly and effectively responded to their client’s crisis notification while they were out of office. They voiced, “It was nice to know that the days you are away or not feeling well… if someone falls down, they’re still going to get caught. They’re still going to have a lifeline, even if you’re not it” (P15). Another participant described how the alerts emphasized the strength and cohesion of their team: “…it was a success because we learned how effective our clinician team was […] …it was a good experiment in how a tool like this could unite clinicians on our team to take quick action” (P07).

Taking into account the advantages to their practice and service setting, some participants perceived a *mission alignment* between the relationship-building capabilities of DMH and the relational values of their organizations. Participants also felt that DMH and associated setting-wide collaboration to meet youth’s needs contributed to their service settings’ culture of *recipient-centeredness*.

### Theme 4: DMH Platform Facilitators

#### Overview

Theme 4 explores facilitators to using DMH with youth experiencing STBs that relate to qualities of DMH itself. This includes subtheme 4.1 (DMH enables disclosure, identification, and early intervention).

#### Subtheme 4.1: “To Be Able to Intervene and Make Sure Someone’s Life Is Safe”: DMH Enables Disclosure, Identification, and Early Intervention

In a majority of interviews, clinicians noted that DMH expanded their existing awareness about youth on their caseloads who experience STBs—and the extent and urgency of their struggles. Information about youth’s STBs gleaned from DMH was considered a strong *relative advantage* even among participants who faced substantial barriers to implementing DMH with youth in crisis. For youth who “barely speak” (P13), “are not forthcoming with that information” (P12), or “would never ever approach suicidality head-on” (P10), DMH created a safe space for disclosure and facilitated identification of STBs by their clinician. Participants also recognized that the STBs domain “discovered or identified” youth whom they “had no knowledge that they were suicidal” (P09), in addition to aiding identification in cases where they were already conscious of possible risk. Some participants were uncomfortable using DMH as their primary means of identification, but appreciated it as a supplementary tool alongside in-person assessment and clinical judgment.

Following disclosure and identification, clinicians stressed another *relative advantage* of DMH: “to be able to intervene and make sure someone’s life is safe” (P29). Participants frequently spoke to the benefits of DMH with regard to early intervention, the provision of timely support, the ability to share internal and external resources, and how this met the *needs* of youth. Sometimes, this included expediting youth on waitlists to receive earlier and more intensive treatment to address their STBs. One participant shared that overcoming clinician discomfort to discuss STBs identified via DMH may prevent later escalation and the need for emergency services:

*I know clinicians are generally scared about suicide, and it’s always uncomfortable. But actually, when you look at it from the perspective of, let’s get in there early and have that conversation. I haven’t needed to send anybody to the hospital, or get any services involved immediately […]. We’ve gone through a suicide [safety] plan […]. It’s allowed for youth to talk through what they could do, what they might do*.

[P05]

Participants also felt that DMH itself had a “big role to play” (P19) in early intervention by providing resources, such as apps and e-tools, that youth can use to self-manage their STBs and reinforce coping skills between appointments.

Finally, some clinicians were optimistic that documentation about STBs in DMH might improve the quality of the youth mental health care system overall. One participant contemplated:

*I think there’s value to measurement-based care […]. The more that we’re able to research, document, the better. It’s actually scary, how often something happens in the medical field that we don’t have proper documentation of, we don’t have proper measurement of. We don’t know that 70 people were suicidal. We have absolutely no idea when, why, circumstances, how suicidal they were… The more information that we have, and can collectively share, is better*.

[P13]

They and other clinicians saw the potential for documentation and resultant system-level improvement as an overarching *relative advantage* of DMH—and ultimately, a gain for young people themselves who require support with their experiences of suicidality.

### Clinician Recommendations

Clinicians often strategized potential solutions to the identified service setting, professional practice, and DMH platform barriers. Their recommendations are provided in Table 4 in accordance with the overarching barrier themes.

**Table 4.** Clinician recommendations for addressing the barriers to using digital mental health (DMH) with youth experiencing suicidal thoughts and behaviors (STBs).

Theme and barrier	Clinician recommendations
Service Setting and Professional Practice Barriers	
Service settings do not support immediate crisis response due to restricted clinic hours.	Service settings implementing DMH^a^ should integrate a designated crisis response professional or crisis response team from within or outside the service setting who are available to respond to youth flagged in DMH. This professional or team should have flexible availability within and outside service setting hours.
Clinicians have limited opportunity to integrate crisis response within their schedules.	Refer to above recommendations.
Clinicians face barriers related to responding to youth in crisis outside working hours, including feeling burdened in their personal time and concerns about liability.	Refer to above recommendations. Outside service setting hours, DMH should notify local and available crisis service settings instead of notifying clinicians.
Clinicians have limited opportunity to respond and are unsure how to respond to youth in crisis that they perceive are “overreporting” their symptoms.	The DMH STBs^b^ assessment should include questions that assess “bigger picture” experiences with STBs, in addition to in-the-moment feelings of distress and suicidality.
DMH Platform Barriers	
Some wording in the DMH STBs assessment can be interpreted multiple ways and lacks context.	Revise wording to make assessment questions clearer and easier for youth to interpret. Clinicians note that youth in crisis are not thinking clearly; ensure that the assessment and crisis resources are easy for youth to navigate.
Some youth may struggle to interpret the DMH STBs assessment and cope with reading and seeing the visual of their results.	Complete assessment with youth in-session; however, clinicians note that this may take additional time.
Youth with persistent or past STBs are continuously flagged as high-risk in the platform.	The assessment should clearly distinguish between past and present STBs. DMH should include an open text box for youth to provide comments and context about STBs (eg, when they occurred—past vs present). DMH should include an open text box for youth to provide follow-up comments and context about STBs (eg, did they access crisis resources and are they still suicidal). If youth are no longer in active crisis and report this, DMH should “un-flag” youth. DMH should show improvement for youth with persistent STBs who are improving yet still require close monitoring.

a DMH: digital mental health.

b STB: suicidal thoughts and behaviors.

## Discussion

### Principal Findings

This study explored clinicians’ perceptions of using DMH with youth experiencing STBs. A theoretical thematic analysis identified barriers and facilitators related to clinicians’ service settings and professional practice, and DMH itself. Our findings highlight important topics for discussion, practical implications and recommendations, and directions for future research.

In the first theme, participants shared that their service settings were not intended to provide immediate and all-hours crisis response to youth who score high for STBs in DMH. Concerns about the compatibility of DMH due to clinic hours and school closures were expressed in preimplementation focus groups conducted by our research and implementation team [58,59]. Similarly, other studies document clinician apprehension about youth reporting their STBs outside working hours [60-62], while a clinician is on leave [61,62], or if a clinician works part-time [61]. Our findings reveal additional fears that clinicians are unable to ascertain that the crisis resources provided in DMH (eg, crisis helplines or community-specific crisis resources) will adequately support youth in their moment of need. If crisis notifications occur during working hours, clinicians’ ability to respond while fulfilling their existing roles and responsibilities is still limited by hectic schedules, competing demands [58], and high-acuity client caseloads [60].

The first theme also describes that clinicians’ deep care for their clients’ well-being extends beyond clinic hours to impact their personal time and thoughts. Although previous studies acknowledge that DMH may contribute to clinician burden [61,62], our findings more clearly define the emotional impact of crisis notifications as a unique barrier to clinicians’ acceptance of DMH. This study restates another preimplementation worry that clinicians may be held legally responsible if crisis notifications are not immediately attended to due to working hours and capacity, and an adverse event occurs [58,59].

A final and noteworthy finding in the first theme is that youth experiencing STBs may under- or overreport their symptoms in DMH. Underreporting or nondisclosure of STBs may relate to fears about confidentiality [40], potential harmful reactions from clinicians [42], or other factors, which may contribute to some youth’s preference for DMH that is self-guided [38]—vs DMH that provides shared access to youth’s data (eg, Innowell). Our study also identifies a marked incongruity between clinicians’ assessment of the criticality of youth’s STBs and youth’s expressed vulnerability and urgency in the DMH STBs domain. As said by one participant, frequent crisis notifications may occur if youth are only comfortable and able to alert clinicians about their STBs through online mediums [52]. Participants’ perception that responding to continuous alerts may reinforce ineffective coping is consistent with Hetrick et al’s [62] finding that clinicians perceive that DMH may unintentionally create learned helplessness.

Of relevance to all concerns in the first theme, 2 studies exploring both clinician and youth perspectives about DMH emphasize youth’s insight into clinician barriers [61,62]. Hetrick et al [62] found that youth were understanding of clinicians’ working hours, the difficulty of responding to crisis notifications after hours, and were aware that crisis resources (eg, crisis helplines) may not guarantee immediate support. Youth participants in a co-design study shared worries that monitoring risk via DMH may be burdensome for clinicians and thus may negatively impact the therapeutic alliance [61]. While mindful of clinicians’ time and capacity, youth did want their clinician to know if they had used crisis resources and received support [62]. Some youth felt that automated protocols should have sufficiently high thresholds for determining the severity of risk to reduce clinician burden [61].

In the second theme, participants perceived the DMH STBs domain’s complex wording and lack of context as barriers to youth using DMH and DMH accurately reflecting youth’s distress. Preimplementation data similarly captured concerns that DMH may be unsuitable for youth experiencing STBs, difficult to navigate, and difficult to interpret [58,59]. This study evidences such concerns manifesting in practice and adds that, in addition to the assessment itself, results may be challenging for youth to understand and cope with without clinician support. Accessibility, ease of use [52,54], and “clear and precise questions” [54] are noted as facilitators to youth with STBs engaging with DMH.

The second theme also describes the sensitivity of the DMH automated suicide escalation protocol and frequency of crisis notifications, contextualized by participants’ acute client populations. It is worth noting that a majority of our participants were rurally located. Rural communities in Canada face a higher burden of mental health crises [12], due in part to inequitable access to mental health resources, stigma, and economic pressures [79,80]. This aligns with our finding that many youth in rural areas experience ongoing STBs or have past STBs—and thereby trigger frequent alerts. Prior work explains how youth seeing their decline in DMH may create feelings of disappointment, shame, guilt, or even the urge to engage in self-harm [50]. Further, a study about best practices for working with youth experiencing STBs [40] supports our participants’ concern that continuous crisis notifications may oppose strengths-based therapeutic approaches. Youth participants were uncomfortable being labeled as at-risk, felt that risk assessment may be reductive, and desired more encouraging assessment language to facilitate help-seeking [40]. Despite this, youth accentuated the importance of talking about STBs with clinicians and wanted clinicians to ask about STBs.

Findings in the third theme demonstrate that DMH can facilitate conversations about STBs between clinicians and youth. Other researchers have addressed the potential for DMH to instigate a collaborative dialogue for youth who have not yet shared their STBs [53], youth who feel ashamed and are therefore reticent to disclose STBs in-person, and youth who are more comfortable expressing themselves online [52]. In turn, conversations about STBs and safety planning may build therapeutic rapport [44,53]. Our study adds that DMH may promote a stronger relationship between youth and the service setting by uniting clinician teams to support youth in distress. In contrast to positive findings, Sundram et al [61] found that some clinicians felt that DMH had no impact on their relationship with youth. Systematic reviews note that DMH may only promote deeper relationships for youth experiencing STBs if it is leveraged as a complement to traditional care, rather than replacing it fully [44,52].

Finally, the fourth theme illustrates the life-saving capacity of DMH by facilitating disclosure, identification, and early intervention for STBs. Disclosure of STBs may be more likely in DMH vs face-to-face assessment given youth’s comfort with technology [45,52,54,60]. As a result, clinicians are better able to detect youth’s urgent needs and understand the breadth of their symptoms [19,53,54,68]. Our finding that DMH supports early intervention is congruent with several studies reporting that DMH improves service settings’ and clinicians’ response to suicide risk by assisting clinical decision-making about youth’s treatment [19,54,57,68] and escalating youth on waitlists to receive immediate care [19,53]. Additionally, previous work indicates that apps and e-tools for STBs (such as those provided in Innowell) may bridge the gap in-between sessions [52]—particularly if clinicians practice using the resources with youth to increase their comfort level [40]. This study also brings to light that information about STBs in DMH may be documented and acted upon to improve youth mental health services and targeted supports for suicidal youth.

### Practical Implications and Researcher Recommendations

The fact that many of clinicians’ preimplementation concerns were realized when using DMH in practice underscores the validity of their apprehension, and the need to hear and respond to their concerns. In relation to service setting and professional practice barriers, we found that adaptations are required to ensure that youth with the courage to share their STBs receive the support that they need. We echo our participants’ recommendation that service settings implementing DMH should designate staff with scheduling availability and flexibility to monitor and respond to crisis notifications in DMH. Service settings might consider adding multiple clinicians or assigning a clinical team as support persons to youth’s DMH profile, as well as including caregivers or other trusted adults as emergency contacts. Automatically alerting all-hours crisis service settings in the community, rather than clinicians, may also alleviate burden outside working hours—while still documenting STBs in DMH for later viewing. Concerns about liability should be addressed by providing clinicians using DMH with sufficient training and understanding of local laws, policies, and standardized clinic procedures for responding to youth in crisis. Along with procedural changes, we call for increased organizational support for clinicians whose own mental health is affected by working with youth experiencing STBs. Access to mental health resources and regular debriefing with clinician peers and leadership may support clinicians in managing stress and vicarious trauma. Regarding youth who underreport their STBs, clinicians should consider DMH as similar to paper-and-pencil assessments and rely upon their clinical judgment and relationship with youth to determine if youth are minimizing their distress. Clinicians should recognize that youth that they perceive are “overreporting” their STBs may not feel that they are exaggerating and may require more intensive treatment. Before using DMH, creating clear expectations of clinicians’ availability and response times to crisis notifications may be helpful so that youth are not distressed by not receiving an immediate response.

To resolve DMH barriers, DMH developers should differentiate crisis notifications for youth who (1) are continuously suicidal or have past STBs, vs (2) youth experiencing active crises requiring urgent support. Reporting ongoing or past STBs could trigger a “secondary” alert that suggests checking in with youth in-session rather than rapid clinical action. Our participants’ recommendation for DMH to include open text boxes may allow youth to comment on assessment questions they are unsure of and add context to their responses. In Sundram et al’s [61] study, youth proposed a higher threshold for alerts and suggested that some youth may be capable of identifying and communicating their level of urgency in DMH.

This study emphasizes the value of implementation frameworks to assess implementation processes, identify DMH-specific barriers, and guide the design and adaptation of DMH itself. For instance, applying frameworks such as the CFIR [74] iteratively throughout DMH development and preimplementation project phases may uncover key contextual factors to address early in design (eg, outer and inner setting context, clinician capacity, and considerations for youth with ongoing or past STBs), improve alignment between DMH features and clinical processes, and increase responsiveness to user needs.

Our findings also offer practical implications for augmenting personalized care using DMH and youth’s data. Considering individual risk factors for STBs and youth’s unique perceptions and experiences, personalization is a facilitator to engaging with DMH [52]. Previous work highlights that multidimensional DMH assessment may detect personal underlying issues impacting STBs [50,53] and track patterns in youth’s thoughts and behaviors [50]. By recognizing contributing factors beyond the symptom itself, DMH may enhance youth’s self-knowledge [50] and facilitate the delivery of personalized care by clinicians [53]. We further suggest that DMH include features that enable youth and clinicians to codevelop a personalized safety plan with coping strategies, apps, e-tools, and emergency contacts.

Aligned with our larger project’s outcome objectives, evidence shows that early intervention for STBs may mitigate hospitalization [81] and suicide attempts [82]. We advocate for the continued implementation of DMH in health care systems and other youth-serving institutions and organizations (eg, schools) to meet youth where they are, identify their areas of need, and intervene for STBs at early stages.

### Future Directions

As digital interventions for suicidality evolve, DMH tools that assess multiple health domains remain fundamental to capturing holistic data and recognizing the multifactorial nature of risk factors for STBs. Research on DMH specific to STBs is another important area for attention and development. At present, novel approaches include (but are not limited to) natural language processing systems that detect STBs [44], real-time monitoring tools that assess STBs at the time they occur [20], machine learning [83] and continuous-time trajectory models [84] that predict STBs, and just-in-time adaptive interventions that provide personalized in-the-moment support [11]. There is a lack of research on DMH for STBs that acknowledges and incorporates aspects of culture [52] and intersectionality. Given the compounded risk factors experienced by some populations (eg, youth who are racialized and/or 2SLGBTQIA+), community co-design of DMH for STBs is a significant and urgent area for growth, and an opportunity to support youth who are underserved and excluded by current health care systems. Trauma-informed DMH apps for youth exposed to trauma and experiencing STBs is another direction for future research. For all types of DMH, the inclusion of youth perspectives and the lived experience lens is imperative to shape accessible, inclusive, and effective resources.

All existing and future DMH developments necessitate rigorous testing and evaluation of implementation in a variety of settings. Our research and implementation team is continuing to implement DMH and share our findings of the perspectives of clinicians [58,59,64], leadership, and young people receiving care [65] to identify obstacles and practical solutions.

### Strengths and Limitations

To our knowledge, this qualitative analysis is one of few studies investigating clinician perceptions of using DMH with youth experiencing STBs. The inclusion of clinicians working in PCNs, specialized mental health services, schools, and community-based organizations in rural and urban locations across Alberta, Canada, provides insights from diverse settings. Clinicians’ ethnic diversity is another strength within our demographics—although we were unable to report specific ethnic origins to protect participants’ identities. During data analysis, the CFIR offered a comprehensive theoretical framework for producing a rich description of themes. The involvement of researchers with lived experience of STBs and who have been affected by youth suicide aided in understanding the complexity of experiences of STBs, receiving treatment, and working with youth as clinicians.

A limitation within our demographics is that most participants identified as women. While the gender distribution is illustrative of mental health clinicians in Canada, the addition of perspectives of men and gender diversity may have enriched our findings. Our relatively small sample size of 32 clinicians in Alberta may not be representative of other communities and contexts. Finally, this paper outlines the perceptions of clinicians only; including youth along with clinicians may have resulted in different findings and recommendations.

### Conclusions

This study sought to identify barriers and facilitators to using DMH with youth experiencing STBs in youth-serving mental health service settings across Alberta, Canada. Clinicians perceived service setting and professional practice barriers, including that the structure and purpose of service settings do not support immediate response to crises flagged in DMH, DMH crisis notifications create tension between clinicians’ responsiveness and the emotional burden of monitoring DMH outside working hours, and clinicians are uncertain how to manage and respond to youth with complex needs who communicate their distress in DMH in differing ways. The complexity of the DMH STBs domain and continuous crisis notifications for youth with persistent or past suicidality were identified as barriers in DMH. Despite their concerns, clinicians saw that using DMH collaboratively with youth supported the therapeutic relationship and aligned with their service settings’ mission and values. Disclosure, identification, and early intervention for STBs enabled by DMH significantly benefited youth and inspired clinicians to continue implementing digital tools.

Given the association between STBs and suicide, attending to STBs is a critical component of suicide prevention. Our findings demonstrate that direct assessment of STBs using DMH may address the youth suicide crisis by facilitating disclosure and rapid clinical action to support early intervention. Moreover, multidimensional assessment may reveal important information about STBs and risk factors, with advantages for personalized care and system-level improvement. Clinicians delivering DMH must be supported by applying their recommendations and continuously strategizing to mitigate barriers and leverage facilitators for sustained implementation.

## Abbreviations

2SLGBTQIA+: Two-Spirit, Lesbian, Gay, Bisexual, Transgender, Queer or Questioning, Intersex, Asexual, + other sexual orientations and gender identities
CFIR: Consolidated Framework for Implementation Research
DMH: digital mental health
MBC: measurement-based care
PCN: primary care network
RA: research assistant
REDCap: Research Electronic Data Capture
STB: suicidal thoughts and behaviors
WHO: World Health Organization
